# Weight Loss in Cancer Patients Correlates With p38β MAPK Activation in Skeletal Muscle

**DOI:** 10.3389/fcell.2021.784424

**Published:** 2021-12-07

**Authors:** Guohua Zhang, Lindsey J. Anderson, Song Gao, Thomas K. Sin, Zicheng Zhang, Hongyu Wu, Syed H. Jafri, Solomon A. Graf, Peter C. Wu, Atreya Dash, Jose M. Garcia, Yi-Ping Li

**Affiliations:** ^1^ Department of Integrative Biology and Pharmacology, University of Texas Health Science Center, Houston, TX, United States; ^2^ Geriatric Research, Education and Clinical Center (GRECC), VA Puget Sound Health Care System, Seattle, WA, United States; ^3^ Department of Medicine, Division of Gerontology and Geriatric Medicine, Seattle, WA, United States; ^4^ Department of Medicine, Section of Oncology, University of Texas Health Science Center, Houston, TX, United States; ^5^ Division of Medical Oncology, University of Washington School of Medicine, Seattle, WA, United States; ^6^ Department of Surgery, University of Washington School of Medicine, Seattle, WA, United States; ^7^ Department of Surgery, Veterans Affairs Puget Sound Health Care System (VAPSHCS), Seattle, WA, United States; ^8^ Department of Urology, Veterans Affairs Puget Sound Health Care System (VAPSHCS), Seattle, WA, United States

**Keywords:** P300, C/EBPbeta, ULK1, UBR2, Hsp70, Hsp90

## Abstract

Unintentional weight loss, a first clinical sign of muscle wasting, is a major threat to cancer survival without a defined etiology. We previously identified in mice that p38β MAPK mediates cancer-induced muscle wasting by stimulating protein catabolism. However, whether this mechanism is relevant to humans is unknown. In this study, we recruited men with cancer and weight loss (CWL) or weight stable (CWS), and non-cancer controls (NCC), who were consented to rectus abdominis (RA) biopsy and blood sampling (n = 20/group). In the RA of both CWS and CWL, levels of activated p38β MAPK and its effectors in the catabolic pathways were higher than in NCC, with progressively higher active p38β MAPK detected in CWL. Remarkably, levels of active p38β MAPK correlated with weight loss. Plasma analysis for factors that activate p38β MAPK revealed higher levels in some cytokines as well as Hsp70 and Hsp90 in CWS and/or CWL. Thus, p38β MAPK appears a biomarker of weight loss in cancer patients.

## Introduction

Unintentional weight loss is a hallmark of cachexia and one of the most challenging aspects of managing cancer patients with no established treatment. It is estimated that weight loss impacts approximately 60% of cancer patients. Cancer-associated weight loss is largely due to muscle mass loss (muscle wasting), and weight loss is a first available sign of muscle wasting in clinics. About half of cancer patients who have suffered weight loss eventually die of it (Baracos et al., 2018). Thus, weight loss is a major threat to cancer survival. However, cancer-induced weight loss is currently inexorable due to the lack of established etiology and treatment.

Muscle wasting is a central component of cancer-associated weight loss and it can be induced by various catabolic stimuli in the cancer milieu through an overactivation of the ubiquitin-proteasome pathway (UPP) and the autophagy-lysosomal pathway (ALP), resulting in accelerated muscle protein degradation. However, unlike muscle atrophy induced by disuse, fasting and denervation, cancer-associated muscle wasting features a severe systemic inflammation that stimulates muscle catabolism. A number of inflammatory cytokines found in the cancer milieu stimulate muscle protein degradation (Baracos et al., 2018). However, clinical trials employing anti-cytokine strategies have not yielded satisfactory results (Yennurajalingam et al., 2012; Molfino et al., 2019). Thus, we sought to identify inflammation-activated intracellular signaling pathways that are central in mediating cancer-induced muscle wasting. We have uncovered in diverse murine cancer models a p38β MAPK—p300—C/EBPβ signaling pathway that is essential for the initiation and progression of muscle wasting. Upon activation by tumor-released catabolic mediators, p38β MAPK activates the transcription factor C/EBPβ through two intricate steps. First, p38β MAPK activates the acetyltransferase activity of p300 by phosphorylating its Ser12 residue, which enables p300 to acetylate C/EBPβ on its Lys39 residue and activates C/EBPβ′s transactivation activity (Sin et al., 2019a). Second, p38β MAPK directly phosphorylates C/EBPβ on its Thr188 residue to activate its binding to DNA (Zhang and Li, 2012). Activated C/EBPβ upregulates key genes in UPP such as *atrogin1* (Zhang et al., 2011) and *UBR2* (Zhang et al., 2013), and in ALP such as *LC3b* and *Gabarapl1* (Liu et al., 2018). In addition, p38β MAPK activates autophagosome formation by directly phosphorylating ULK1 on its Ser555 residue (Liu et al., 2018). These events lead to increased degradation of muscle proteins such as myosin heavy chain, a target of UPP, and p62, a target of ALP, respectively, resulting in muscle wasting (Liu et al., 2018). Consequently, deleting p38β MAPK (Liu et al., 2018), p300 (Sin et al., 2019a) or C/EBPβ (Zhang et al., 2011) individually abrogates muscle wasting in tumor-bearing mice. In addition, pharmacological inhibition of the three signaling molecules individually alleviates muscle wasting in tumor-bearing mice (Zhang et al., 2011; Sun et al., 2016; Sin et al., 2019a). Most recently, we showed that selectively inhibiting p38β MAPK using the kinase inhibitor nilotinib approved for chronic myelogenous leukemia alleviates muscle wasting in tumor-bearing mice (Sin et al., 2021). Thus, we ask the question whether p38β MAPK is relevant to cancer-induced muscle wasting and weigh loss in humans.

In addition, we previously identified cancer-released circulating Hsp70 and Hsp90 as essential mediators of muscle wasting in preclinical studies (Zhang et al., 2017a). Murine and human cancer cells including lung, pancreatic, colon and gastric cancer constitutively release high levels of Hsp70 and Hsp90 as surface proteins on extracellular vesicles (EVs), which induce muscle wasting by directly activating TLR4 on muscle cells. In parallel, elevated circulating Hsp70 and Hsp90 cause elevation of circulating inflammatory cytokines such as TNFα and IL-6 through the systemic activation of TLR4 (Zhang et al., 2017a). TLR4 mediates cancer-induced muscle wasting through p38β MAPK-mediated muscle protein loss (Zhang et al., 2017b). As a common effector of TLR4 and inflammatory cytokine receptors that promote muscle wasting including TNFα (Li et al., 2005), IL-6 (Hetzler et al., 2015), IL-1β (Li et al., 2009) and members of the TGFβ superfamily including TGFβ (Waning et al., 2015) and activin A/myostatin (Ding et al., 2017; Zhong et al., 2019), p38β MAPK plays a central role in mediating muscle wasting in murine models of cancer (Sin et al., 2019b; Sin et al., 2021). However, whether circulating Hsp70 and Hsp90 elevate in association with p38β MAPK activation in cancer patients with weight loss is unknown.

We recruited patients who underwent abdominal surgery to remove diverse types of cancer in the gastrointestinal (GI) or genitourinary (GU) tract with or without weight loss, for rectus abdominis (RA) muscle biopsy and plasma analyses to compare with non-cancer patients without weight loss who underwent laparotomy for other reasons. We found that in these cancer patients, higher p38β MAPK activity co-exists with increased catabolic signaling, lower levels of muscle proteins and elevated circulating Hsp70 and Hsp90. Particularly, p38β MAPK activation correlates with weight loss. These data suggest that p38β MAPK is a biomarker of weight loss in cancer patients.

## Patients and Methods

### Patients

A single-center, cross-sectional patient study was conducted at the Veterans Affairs Puget Sound Health Care System (VAPSHCS) in Seattle, WA, United States between March 2016 and June 2019. This protocol was approved in advance by the VAPSHCS Institutional Review Board and the Research and Development Committee and was conducted in compliance with the Declarations of Helsinki and its amendments and the International Conference on Harmonization Guideline for Good Clinical Practices.

Sixty male subjects who were scheduled for abdominal surgery were recruited and divided into three groups of 20 subjects each: 1) cancer with weight loss (CWL), having cancer of the gastrointestinal (GI) or genitourinary (GU) tract and an involuntary weight loss of at least 5% over the previous 6 months, 10% over the previous 12 months or 2% over the previous 6 months with a BMI < 20; 2) cancer with weight stable (CWS), having cancer of the GI or GU tract but no significant weight loss; and C) non-cancer control (NCC), having no active cancer (non-melanoma skin cancer was an allowable comorbidity, if present) or significant weight loss within the last 5 years. Exclusion criteria included other causes of unintended weight loss such as congestive heart failure, liver disease, renal failure, active/uncontrolled infection, uncontrolled diabetes mellitus (HbA1c ≥ 9%), active users of an anabolic or investigational agent, user of high dose steroids (20 mg of prednisone/day for more than 1 month), or megestrol.

After signing the informed consent and review of inclusion and exclusion criteria, height and body weight were measured on a calibrated scale dedicated for this study and body mass index (BMI) was calculated. A fasting blood sample was taken and stored as plasma at −80°C. During clinically indicated laparotomy, rectus abdominis (0.5–1 g) biopsies were collected via transverse incision and sharp dissection. Specimens were quickly, grossly dissected if any cauterized edges or vascular tissue were visualized, then immediately flash frozen using liquid nitrogen and were stored at −80° until analysis or stored in RNA-Later for RNA analysis (Anoveros-Barrera et al., 2019). Collected samples were shipped to University of Texas Health Science Center at Houston, where the samples were analyzed in a blinded fashion.

### Western Blot Analysis

Western blot analysis of muscle homogenate was carried out as previously described (Zhang et al., 2017a). Optical density of individual protein bands was normalized to loading control vinculin or the corresponding total protein in the case of phospho-proteins, which was shown in arbitrary unit (AU). Information on the source, dilution and sample loading of commercially obtained primary antibodies is presented in Supplementary Table S1. Custom-made antibodies for Lys39-acetylated C/EBPβ (1:2,000, 10 μg loading) (Sin et al., 2019; Sin et al., 2021), Ser12-phosphorylated p300 (1:1,000, 30 μg loading) and atrogin1/MAFbx (1:1,000, 20 μg loading, specificity verified in Supplementary Figure S1) were generated by Pocono Rabbit Farm & Laboratory.

### Quantitation of mRNA

Real-time PCR was performed as described previously (Zhang et al., 2017a). Sequences of specific primers are UBR2 (sense: 5′-cag​gac​tgg​tgt​gct​tca​ga -3′, antisense: 5′-TGT​GAT​TGG​CTG​TTC​ACC​AT -3′); atrogin1/MAFbx (sense: 5′-GGC​TGC​TGG​AAG​AAA​CTC-3′, antisense: 5′-CCT​TCC​AGG​AAA​GGA​TGT​GA-3′); MuRF1 (sense: 5′-CCC​ATG​GAG​AAC​TTG​GAG​AA-3′, antisense: 5′-AGC​CTG​GAA​GAT​GTC​ATT​GG-3′); LC3b (sense: 5′-CTG​TTG​GTG​AAC​GGA​CAC​AG -3′, antisense: 5′-CTG​GGA​GGC​ATA​GAC​CAT​GT -3′); gabarapl1 (sense: 5′-GGT​CCC​CGT​GAT​TGT​AGA​GA -3′, antisense: 5′-GGA​GGG​ATG​GTG​TTG​TTG​AC -3′); TLR4 (sense: 5′-TGA​GCA​GTC​GTG​CTG​GTA​TC-3′, antisense: 5′-CAG​GGC​TTT​TCT​GAG​TCG​TC-3′); and vinculin (sense: 5′-gttcavaatgcccagaacct-3′, antisense: 5′-TCT​TTC​TAA​CCC​AGC​GCA​GT-3′).

### Quantification of Plasma Cytokines

Patient plasma was analyzed for specific cytokines utilizing Bio-Plex^®^ Pro Human Cytokine Screening Panel (#12007283, Bio-Rad Laboratories, Hercules, CA, United States) according to manufacturer’s protocol using the Bio-Plex 200 reader (software version 6.0, Bio-Rad Laboratories). Raw data were subjected to Gaussian distribution test using the Graphpad Prism 7.00 Software and outliers were excluded.

### Quantification of Plasma Hsp70 and Hsp90

Hsp70 and Hsp90α levels in patient plasma were analyzed by enzyme-linked immunosorbent assay (ELISA) kits from Enzo Life Sciences (#ADI-EKS-715 and #ADI-EKS-875, Farmingdale, NY) following the manufacturer’s instruction.

### Statistical Analysis

Patient variables were summarized by group using N, mean and standard deviation. Data analyses were performed using ANOVA for continuous variables and chi-square for categorical variables.

Correlation was analyzed with Spearman correlation. A *p* value smaller than 0.05 is considered significant.

## Results

Subject characteristics are shown in Table 1. Age, ethnicity, pre-illness body weight and proportion of subjects with a gastrointestinal (GI) and genitourinary (GU) ailment was similar among the groups. The CWL group had significantly greater weight loss over the previous 6 and 12 months. There were no significant differences in tumor types, stage, prior exposure to chemotherapy or radiation and proportion of subjects with metastatic disease between the two cancer groups.

**TABLE 1:** Subject Characteristics. Reasons for surgery in group NCC (n): benign prostatic hypertrophy (Sin et al., 2021), benign colonic tumor (Zhang and Li, 2012), Colonic diverticulitis (Sin et al., 2019a), abdominal hernia (Baracos et al., 2018).

	NCC (*n* = 20)	CWS (*n* = 20)	CWL (*n* = 20)	*p* Value
Age (yrs)	65 ± 8.6	64.9 ± 9.2	65.8 ± 6.8	0.93
Ethnicity (W, B, H, O) (n)	13, 4, 1, 2	14, 2, 1, 3	18, 1, 0, 1	0.62
Body weight (kg)	91.1 ± 13.9	101.2 ± 20.1^a^	84.7 ± 25	0.04
BMI	29.3 ± 4.6	32.5 ± 5.5^a^	26.8 ± 6.4	0.007
Pre-illness weight (kg)	92.4 ± 15	101 ± 20.9	93.7 ± 26.2	0.4
6 mo weight change (%)	−1.29 ± 3.4	0.1 ± 2.3^a^	−8.8 ± 6.8^b^	<0.001
12 mo weight change (%)	−2.3 ± 3.4	−0.5 ± 2.8^a^	−8.8 ± 8.1^b^	<0.001
Organ System (GI, GU), (n)	10, 10	10, 10	10, 10	0.99
Mets (n)	NA	1	3	0.15
Tumor type	NA	8 CRC, 5 PCa, 4 RCR, 2 Ga, 1 Bla	6 CRC, 4 PCa, 4 RCR, 2 Ga, 2 Es	0.74
Stage (I, II, III, IV)	NA	8, 4, 7, 1	3, 10, 4, 3	0.20
Chemotherapy in the previous 3 m (n)	NA	4	3	0.63
Radiotherapy in the previous 3 mo	NA	1	1	NS

Abbreviations: W, White-non-Hispanic; , B, Black non-Hispanic; H, hispanic; O, other; CRC, colorectal cancer; PCa, prostate cancer; RCR, renal cell carcinoma; Ga, gastric adenocarcinoma; Es, esophageal carcinoma; Bla, Bladder cancer.

a Denotes *p* < 0.05 between group CWS and group CWL.

b Denotes *p* < 0.05 in pair-wise analysis compared to group NCC.

**FIGURE 1:**
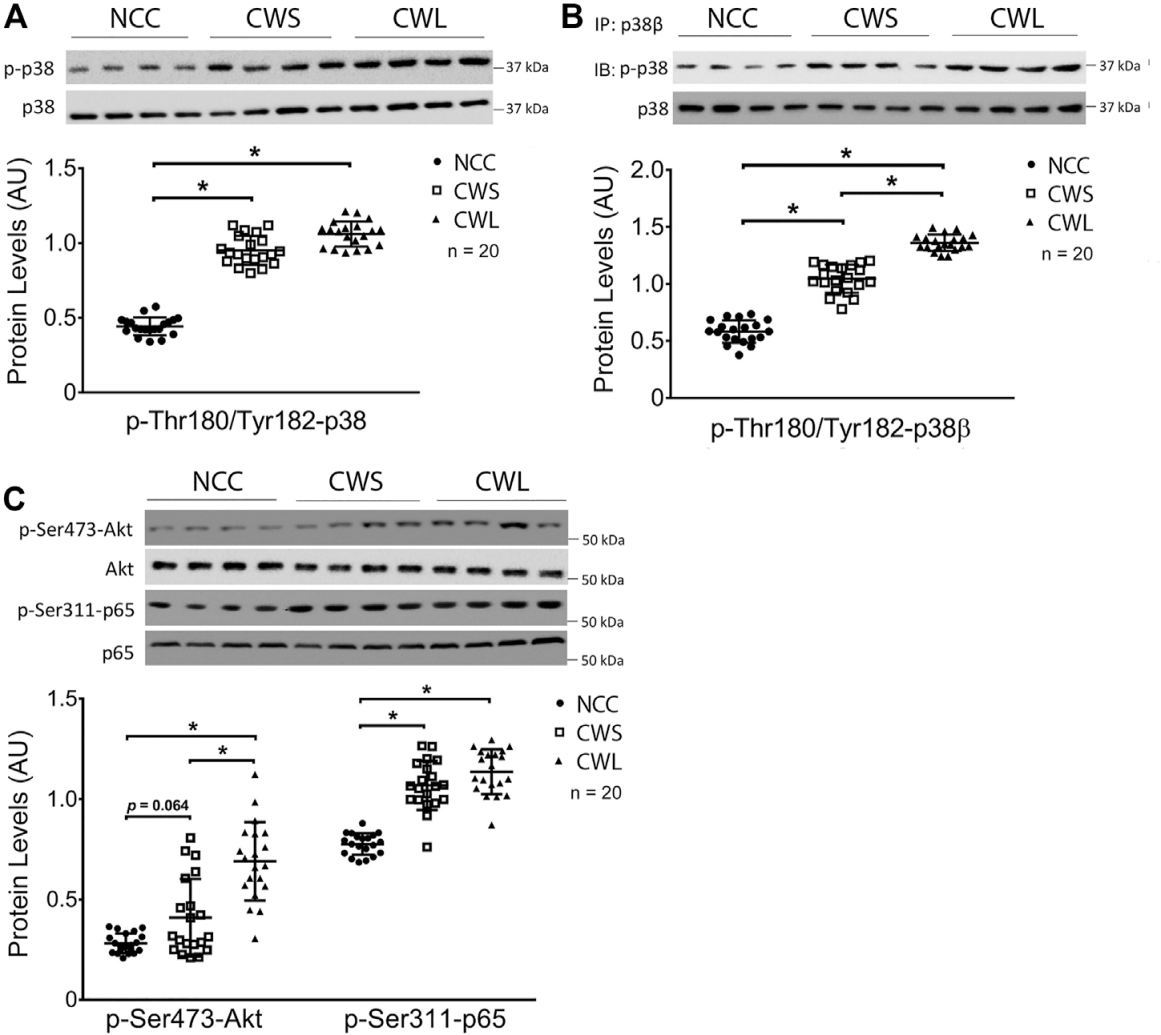
Active pan-p38 MAPK, p38β MAPK, AKT and NF-κB are higher in RA of cancer patients. **(A)** Active pan-p38 MAPK was analyzed by Western blotting. **(B)** Active p38β MAPK was analyzed by immunoprecipitation. **(C)** Active AKT and NF-κB were analyzed by Western blotting. Data were analyzed by one-way ANOVA combined with Tukey’s test. * denotes *p* < 0.05.

Selective signaling proteins implicated in muscle wasting were determined by Western blot analysis of patient RA homogenates. First, higher levels of active pan-p38 MAPK (phosphorylated on Thr-180 and Tyr-182) were detected in CWS and CWL over NCC (Figure 1A). Next, the active form of p38β MAPK was analyzed. Due to the lack of an antibody specific for active p38β MAPK, it was immuno precipitated using a p38β MAPK-specific antibody and its activation was determined by Western blot analysis using the antibody against active pan p38 MAPK. Higher active p38β MAPK levels were detected in CWS, which was further increased in CWL (Figure 1B), suggesting that p38β MAPK is preferentially activated over other members of the p38 MAPK family in cancer patients with weight loss. We also examined the levels of active NF-κB and AKT, which are also TLR4 effectors (Doyle et al., 2011; Zhang et al., 2017b) and often activated in skeletal muscle of cancer patients (Zhou et al., 2010; Stephens et al., 2015). Active AKT (phosphorylated on Ser-473) (Franke et al., 1997) was higher in CWL only while active NF-κB measured as its p65 subunit phosphorylated on Ser-311 (Duran et al., 2003) was higher in both CWS and CWL (Figure 1C).

**FIGURE 2:**
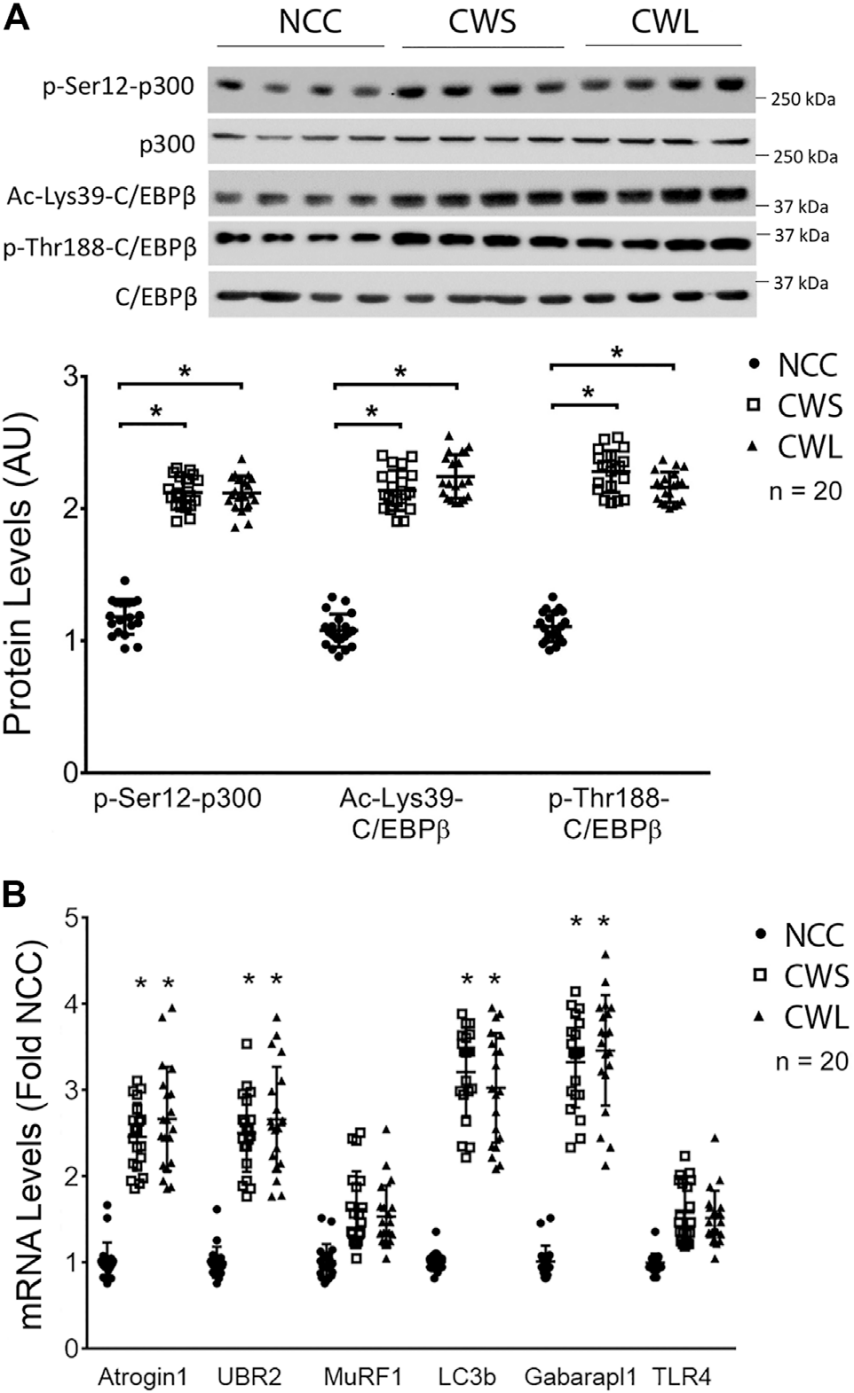
Higher activity of p38β MAPK effectors p300 and C/EBPβ are accompanied by increase in expression of C/EBPβ-responsive genes in UPP and ALP in RA of cancer patients. **(A)** Active p38β MAPK effectors p300 and C/EBPβ were analyzed by Western blotting. **(B)** The mRNA levels of C/EBPβ-responsive genes in the UPP and ALP were analyzed by qPCR. The mRNA of TLR4 was also monitored. Data were analyzed by one-way ANOVA combined with Tukey’s test. * denotes *p* < 0.05 compared to control.

Next, higher levels of active p300 that is phosphorylated on Ser-12 by p38β MAPK (Sin et al., 2021), and active C/EBPβ that is acetylated on Lys-39 by p300 (Zhang et al., 2011) were detected in both CWS and CWL. In addition, higher level of C/EBPβ phosphorylation on Thr-188, mediated by p38β MAPK (Zhang and Li, 2012), was observed in CWS and CWL (Figure 2A). These data suggest that the p38β MAPK—p300—C/EBPβ signaling pathway that mediates muscle wasting in tumor-bearing mice is similarly activated in the muscle of GI and GU cancer patients. To determine whether the higher activity of the p38β MAPK–p300—C/EBPβ signaling pathway is accompanied by higher levels of expression of C/EBPβ-responsive genes in the UPP and the ALP, the mRNA levels of specific genes in patient RA samples were analyzed using qPCR. Higher levels of the mRNA of E3s *atrogin1/MAFbx* and *UBR2* were observed in both CWS and CWL (Figure 2B)*.* On the other hand, expression of non*-*C/EBPβ-regulated *MuRF1* was not different in the three groups. In addition, the mRNA of *Atg8* orthologs *LC3b* and *Gabarapl1* were higher in CWS and CWL (Figure 2B). These data demonstrate that as in tumor-bearing mice (Liu et al., 2018; Sun et al., 2016; Sin et al., 2021), expression of C/EBPβ-controlled genes in UPP and ALP are higher in the skeletal muscle of the cancer patients. We further observed that *TLR4* mRNA was not altered in RA of our cancer patients (Figure 2B), which is consistent to preclinical data that cancer activates TLR4 in muscle but does not alter TLR4 expression (Zhang et al., 2017b).

**FIGURE 3:**
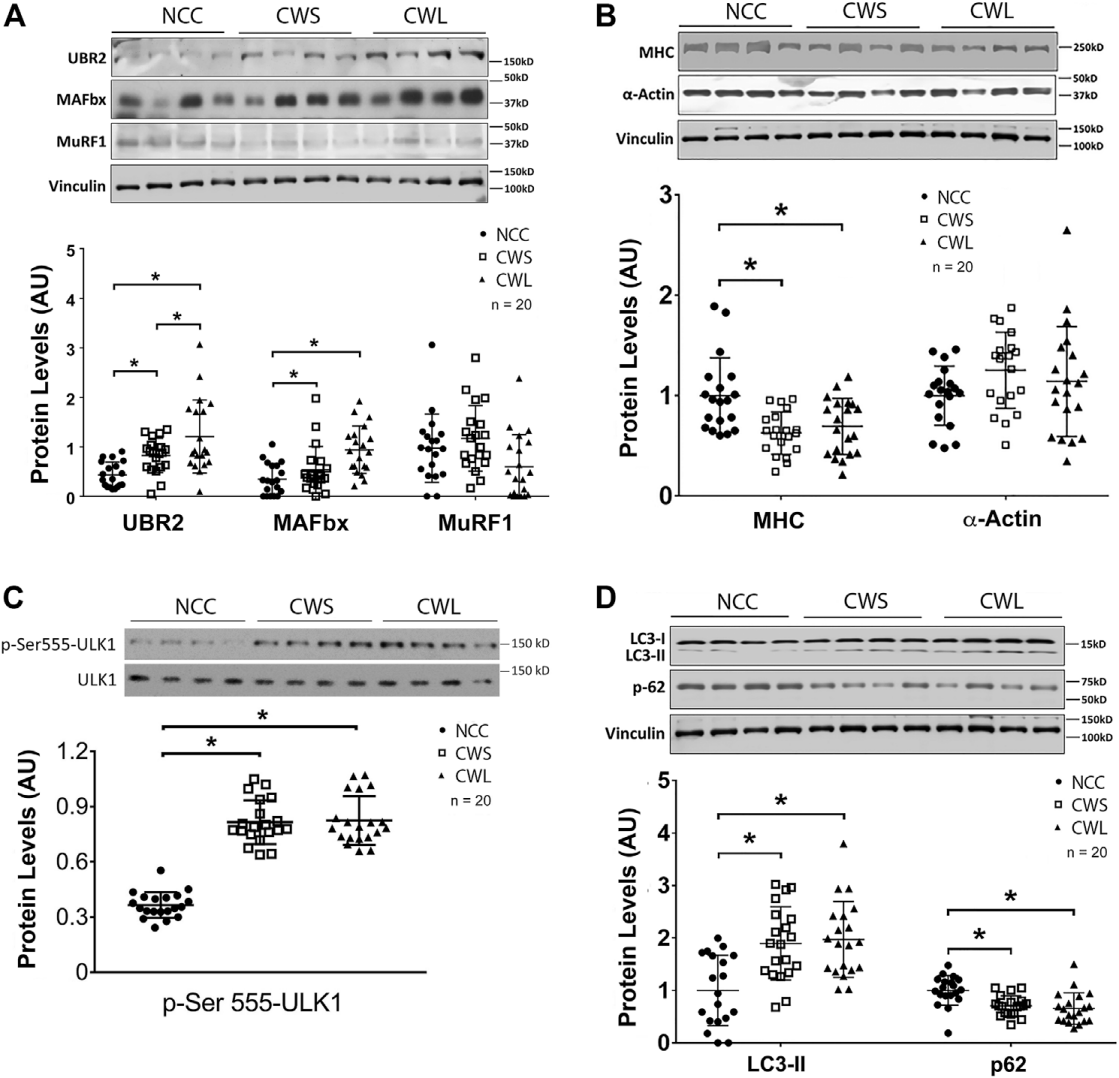
Higher levels of UBR2 and MAFbx as well as active ULK-1 and LC3-II are accompanied by lower myosin heavy chain and p62 levels in RA of cancer patients. **(A)** The protein levels of UBR2, atrogin1/MAFbx and MuRF1 were determined by Western blot analysis. **(B)** Levels of myofibrillar proteins MHC and α-actin were determined by Western blot analysis. **(C)** Active ULK1 was determined by Western blot analysis of its phosphorylation of Ser-555 residue. **(D)** Autophagy activity was determined by Western blot analysis of LC3 and p62. Data were analyzed by one-way ANOVA combined with Tukey’s test. * denotes *p* < 0.05.

To assess whether higher expression of C/EBPβ-controlled genes in UPP led to higher expression of their gene products, the protein levels of C/EBPβ-regulated E3s in RA homogenates were examined. UBR2 level was higher in CWS, and further higher in CWL, while atrogin1/MAFbx level was similarly higher in CWS and CWL. On the other hand, MuRF1 levels remained constant in the three groups (Figure 3A). To assess UPP-mediated protein degradation, we measured the known UPP target myosin heavy chain (MHC) (Cohen et al., 2009) and found that MHC level was at least 35% lower in both CWS and CWL. However, another UPP-targeted myofibrillar protein α-actin were not different among the groups (Figure 3B). In evaluating autophagy activity in the muscle samples a significant higher level of active ULK1 that was phosphorylated on Ser555 was observed in CWS and CWL (Figure 3C). Consequently, autophagy marker LC3-II was higher and autophagy receptor p62 was lower (Figure 3D). These data verify that higher activity of the p38β MAPK—p300—C/EBPβ signaling pathway accompanies higher activity of UPP and ALP, as well as loss of specific muscle proteins that are substrates of UPP and ALP in these cancer patients.

**FIGURE 4:**
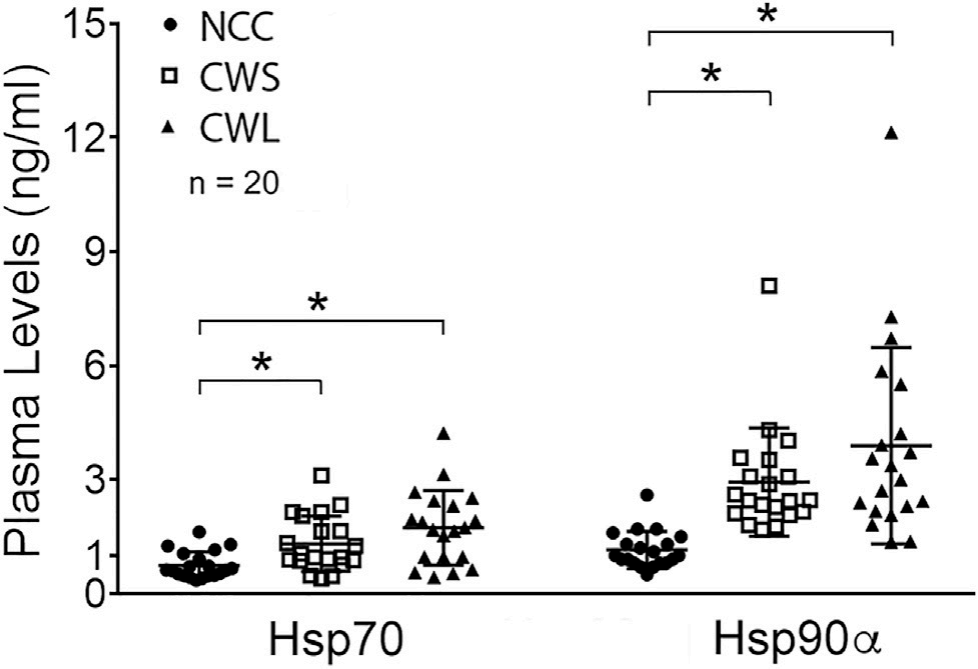
Higher levels of plasma Hsp70 and Hsp90α in cancer patients. Patient plasma Hsp70 and Hsp90α levels were determined by ELISA. Data were analyzed by one-way ANOVA combined with Tukey’s test. * denotes *p* < 0.05.

Higher levels of circulating cytokines can contribute to the increase in active p38β MAPK, NF-κB and AKT (Li et al., 2005; Li et al., 2009; Li et al., 1998). To assess the humoral factors that may activate p38β MAPK in cancer patients their plasma was analyzed for the levels of multiple cytokines using a multiplex panel. As shown in Table 2, higher levels in TNFα, IL-1Rα, MCP-1, IL-8, IL-10 and IL-17 were detected in CWS and/or CWL, while IL-1β level was lower. Further, we measured patients’ plasma Hsp70 and Hsp90α levels using ELISA. Significantly higher levels of plasma Hsp70 (77%) and Hsp90α (157%) were detected in CWS, and a statistically insignificant trend of further higher levels of Hsp70 (133%) and Hsp90α (241%) were observed in CWL (Figure 4). These data recapitulate in cancer patients the elevation of circulating Hsp70 and Hsp90 as well as specific cytokines previously observed in tumor-bearing mice, which promotes muscle wasting at least partially through activating p38β MAPK (Zhang et al., 2017a).

**TABLE 2:** Levels of specific circulating cytokines in cancer patients.

Cytokines	Mean ± SD (pg/ml)			*p* value
	NCC	CWS	CWL	
IFN-γ	42.89 ± 25.72 (*n* = 15)	40.92 ± 17.28 (*n* = 20)	42.85 ± 18.76 (*n* = 20)	0.9461
IL-1α	6.48 ± 1.67 (*n* = 15)	6.74 ± 2.22 (*n* = 19)	8.06 ± 3.64 (*n* = 20)	0.1796
IL-1β	2.28 ± 1.12 (*n* = 16)	2.14 ± 0.43 (*n* = 18)	1.59 ± 0.66 (*n* = 18)^a^ ^,^ ^b^	0.0265^c^
IL-1Rα	224.76 ± 67.73 (*n* = 16)	343.74 ± 131.11 (*n* = 19)^a^	337.93 ± 188.36 (*n* = 19)^a^	0.0286^c^
IL-6	7.85 ± 5.48 (*n* = 14)	7.56 ± 7.34 (*n* = 18)	8.24 ± 5.97 (*n* = 19)	0.9482
IL-8	6.85 ± 2.06 (*n* = 17)	8.79 ± 2.95 (*n* = 19)^a^	10.05 ± 3.43 (*n* = 19)^a^	0.0066^c^
IL-10	1.81 ± 1.05 (*n* = 14)	2.83 ± 1.16 (*n* = 17)^a^	2.8 ± 1.34 (*n* = 14)^a^	0.0407^c^
IL-17	2.09 ± 1.35 (*n* = 16)	2.39 ± 0.70 (*n* = 18)	3.26 ± 1.16 (*n* = 20)^a^ ^,^ ^b^	0.0058^c^
IL-18	75.16 ± 27.77 (*n* = 16)	107.64 ± 47.19 (*n* = 18)	94.71 ± 42.11 (*n* = 20)	0.0726
LIF	21.39 ± 18.41 (*n* = 16)	26.67 ± 14.09 (*n* = 15)	26.20 ± 16.39 (*n* = 18)	0.6063
MCP-1	49.98 ± 20.16 (*n* = 17)	82.44 ± 39.82 (*n* = 20)^a^	57.09 ± 17.00 (*n* = 18)^b^	0.0022^c^
RANTES	2,315.21 ± 1,117.09 (*n* = 15)	3,353.62 ± 2,557.66 (*n* = 17)	2,529.04 ± 1,152.67 (*n* = 18)	0.2111
TNF-α	31.17 ± 9.26 (*n* = 17)	41.64 ± 12.52 (*n* = 19)^a^	36.08 ± 10.43 (*n* = 19)	0.0206^c^
TNF-β	166.08 ± 46.20 (*n* = 17)	173.26 ± 53.47 (*n* = 20)	183.10 ± 55.94 (*n* = 20)^a^	0.612

Data were analyzed by one-way ANOVA combined with Tukey’s test.

a Denotes *p* < 0.05 as compared with NCC.

b Denotes *p* < 0.05 as compared with CWS.

c Denotes *p* < 0.05.

**FIGURE 5:**
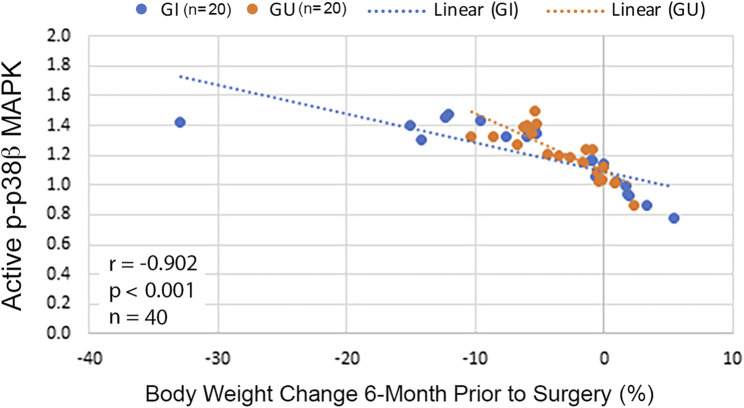
Activity of p38β MAPK in RA correlates with body weight change in cancer patients. Scatterplots with lines of best fit (dotted line) were drawn separately for cancer patients with GI or GU cancer displaying association between p-p38β MAPK level and relative body weight change 6-month prior to surgery. Correlation was analyzed with Spearman correlation for all patients (shown in the figure) and separately for GI (*r* = −0.91, *p* < 0.001, *n* = 20) or GU cancer (*r* = −0.84, *p* < 0.001, *n* = 20).

Because p38β MAPK activity was progressively higher in CWL than CWS, whether p38β MAPK activation correlates with weight loss in our cohort was analyzed with the Spearman correlation test. An inverse correlation between p38β MAPK activation and body weight change was detected in these patients either in total or grouped by cancer type (Figure 5). This data indicates that cancer patients with greater weight loss history had higher p38β MAPK activity. Thus, p38β MAPK appears highly sensitive to the tumor burden and a biomarker of muscle wasting in cancer patients.

## Discussion

The current study provides the first evidence for the clinical relevance of p38β MAPK activation in the pathogenesis of muscle wasting associated with cancer in humans. The finding that p38β MAPK activity inversely correlates with body weight change suggests an important role for this kinase in weight loss associated with GI and GU cancer. Further, higher levels of circulating Hsp70 and Hsp90, in addition to specific cytokines, accompany the activation of p38β MAPK that is a downstream effector of many of these humoral factors. These data are consistent to previous findings from murine models of cancer that in response to TLR4 activation by elevated circulating Hsp70/90 and ensuing elevation of circulating inflammatory cytokines, activated p38β MAPK plays a central role in mediating muscle protein loss that contributes to weight loss induced by diverse types of cancer (Sin et al., 2019b).

We recruited patients indicated for abdominal surgeries with newly diagnosed cancer of GI or GU tract, due to the high prevalence of weight loss in these types of cancer (Baracos et al., 2018) and the easy access to RA muscle during abdominal surgery. In addition, because these patients were newly diagnosed and only 22.5% had received chemotherapy or radiotherapy, the effect of cancer treatments on weight loss was limited. Because stage, tumor type and prior history of cancer treatment were similar in CWS and CWL, the observed differences in body weight between the two groups were unlikely due to these factors. Given that patients with other potential catabolic conditions were excluded from this study, the observed catabolic activity was likely intrinsic to these types of cancer. We observed activation of p38β MAPK-mediated catabolic signaling pathways and muscle protein loss in both CWS and CWL groups. These finding are consistent to previous reports that skeletal muscle catabolism occurs in both weight stable and weight loss patients with lung (Cohen et al., 2009) or GI (Roeland et al., 2017) cancer. These data suggest that skeletal muscle catabolism starts before significant weight loss, and with the progression of the underlying cancer weight loss takes place eventually. Most of our patients were in stage I, II or III of cancer, as cancer progresses patients in the CWS group may develop weight loss over time. Although it is ideal to have muscle mass information to directly quantify muscle wasting, it is very common in clinical settings weight loss is the only available indication of muscle wasting. Our data suggest that p38β MAPK activation is associated with muscle wasting that contributes to weight loss.

We observed that p38β MAPK in skeletal muscle is preferentially activated over total p38 MAPK in the CWL group. We also observed higher activity of p38β MAPK-regulated catabolic signaling molecules including p300, C/EBPβ, and ULK1 as well as upregulation/activation of their respective downstream effectors including UBR2, atrogin1/MAFbx and LC3 in CWS and CWL. However, except UBR2 there was no further increase of those in CWL over CWS. Such differences between p38β MAPK and its downstream effectors is not inconsistent to the likelihood that effect of p38β MAPK activation on muscle catabolism is the sum of its activation of multiple signaling pathways we examined. It is also possible that p38β MAPK activates additional unidentified signaling pathways that contribute to the muscle wasting. Nevertheless, our data reveal that p38β MAPK activation is a very sensitive indicator of and correlates to weight loss in these cancer patients, suggesting that p38β MAPK is a biomarker of cancer-induced muscle wasting.

Myosin loss is a key feature of cancer-associated muscle wasting (Acharyya et al., 2004). MuRF1 directly targets myofibrillar proteins including myosin and α-actin for ubiquitylation and degradation by the 26S proteasome (Clarke et al., 2007; Cohen et al., 2009; Polge et al., 2011). However, MuRF1 is unlikely responsible for MHC loss in our patients because the mRNA and protein levels of MuRF1 remained the same in all three groups while MHC was lower in cancer groups. Such unresponsiveness of MuRF1 mRNA and protein to a cancer burden was previously observed in cachectic cancer patients (Gallagher et al., 2012; Stephens et al., 2010; D'Orlando et al., 2014; Op den Kamp et al., 2013), despite observations of MuRF1 upregulation in some murine cancer models (Zhou et al., 2010; Roberts et al., 2013; Talbert et al., 2014). Given that MuRF1 expression is upregulated by NF-κB (Cai et al., 2004) and down-regulated by AKT (Stitt et al., 2004), the observed increase in active NF-κB and AKT may have negated their individual actions on MuRF1 expression. On the other hand, E3 proteins atrogin1/MAFbx and UBR2 were higher in both groups of cancer patients with UBR2 further higher in CWL, suggesting a significant role of UBR2 in cancer-induced muscle wasting. Atrogin1/MAFbx upregulation indirectly causes MHC loss *via* suppressing its expression (Liu et al., 2018). UBR2 substrates in skeletal muscle cells have not been identified and should be explored in future studies. Interestingly, our data indicate that cancer specifically reduced MHC without affecting α-actin. Given that α-actin is a substrate of MuRF1 (Polge et al., 2011), the absence of MuRF1 response to cancer is consistent with the resistance of α-actin to cancer-induced muscle wasting.

Autophagy activation observed previously in tumor-bearing mice (Liu et al., 2021; Sin et al., 2021) was recapitulated in cancer patients including increase of *LC3b* and *Gabarapl1* as well as active ULK-1, which were accompanied by reduced p62 as expected. These data support that p38β MAPK mediates activation of autophagy in skeletal muscle of these cancer patients.

Under normal circumstances, as an anabolic mediator AKT activation inhibits muscle protein degradation by inactivating FoxO1/3, which suppresses UPP through down-regulating atrogin1/MAFbx and MuRF1 as well as ALP through down-regulating autophagy-related genes including *LC3b* and *Gabarapl1* (Zhao et al., 2007). However, in our cancer patients despite increased active AKT, levels of *atrogin1/MAFbx, LC3b* and *Gabarapl1* were higher and levels of MHC and p62 were lower, while *MuRF1* expression remained same in the three groups. The inconsequence of higher level of active AKT in cancer patients supports a dominant role of p38β MAPK in mediating cancer-induced muscle wasting.

We detected higher levels of TNFα, IL-1Rα, MCP-1, IL-8, IL-10 and IL-17 in the plasma of cancer patients. These cytokines, except IL-10, have been linked to weight loss and muscle atrophy (Baracos et al., 2018). Production/secretion of TNFα (Zhang et al., 2017b), MCP-1 (Wang et al., 2019) and IL-17 (Stuelten et al., 2010) are mediated by TLR4. Notwithstanding, the level of elevation of the individual cytokines seems modest. Particularly, some cytokines implicated in cancer-associated muscle wasting such as IL-6 and IL-1β are not elevated, consistent to a previous study in pancreatic cancer patients (Talbert et al., 2018). Nevertheless, synergy among the elevated cytokines may contribute to the increase of active p38β MAPK. The robust elevation of circulating Hsp70 and Hsp90 in CWS and CWL would activate TLR4 to cause muscle wasting and systemic inflammation (Zhang et al., 2017a). TLR4 plays a major role in mediating cancer-induced activation of p38β MAPK, NF-κB and AKT in skeletal muscle cells (Zhang et al., 2017a; Zhang et al., 2017b). Observed higher levels of active p38β MAPK, NF-κB and AKT in the RA of cancer patients are consistent with TLR4 activation by elevated circulating Hsp70 and Hsp90. We observed higher Hsp70 and Hsp90 levels in CWS, and a trend of further increase in circulating Hsp70 and Hsp90 in CWL. As a progressive process, the development of muscle wasting may be influenced by the degree of elevation of circulating Hsp70/90. More studies are needed to establish the relationship between these markers and weight loss. Particularly, whether circulating Hsp70/90 are biomarkers of cancer-induced weight loss that increase over the disease process should be the focus of future, larger longitudinal studies.

The Hsp70/90—TLR4—p38β MAPK—p300—C/EBPβ signaling pathway is activated in diverse types of murine cancer models including Lewis lung carcinoma (Zhang et al., 2011; Zhang and Li, 2012; Zhang et al., 2013; Zhang et al., 2017a; Zhang et al., 2017b; Liu et al., 2018; Sin et al., 2019a), *Apc* ^*min/+*^ mice bearing intestinal adenocarcinoma (Zhang et al., 2017a) and nude mice bearing human AsPC-1 pancreatic ductal adenocarcinoma (Yang et al., 2019). The current study confirms a higher activity of this signaling pathway in patients of diverse types of GI and GU cancer. Thus, activation of this pathway could be a common mechanism for muscle wasting associated with various types of catabolic cancer.

Taken together, despite the limitation of the current study due to its sectional nature, the data largely recapitulate previous findings on the role of p38β MAPK in mediating cancer-induced muscle wasting derived from murine models (Zhang et al., 2011; Zhang and Li, 2012; Zhang et al., 2013; Zhang et al., 2017a; Zhang et al., 2017b; Liu et al., 2018; Sin et al., 2019a; Yang et al., 2019; Sin et al., 2021). We hereby propose a signaling mechanism that mediates muscle wasting in human cancer as illustrated in Supplementary Figure S2. Validation of this signaling pathway in cancer patients may enable the design of interventions for cancer-associated muscle wasting by targeting p38β MAPK and other signaling molecules in this pathway.

## Data Availability

The original contributions presented in the study are included in the article/Supplementary Material, further inquiries can be directed to the corresponding authors.
