# Supplementary material for: Weight Loss in Cancer Patients Correlates With p38β MAPK Activation in Skeletal Muscle
Source: Front Cell Dev Biol. 2021 Dec 7;9:784424. doi: 10.3389/fcell.2021.784424 (PMC8688918; doi:10.3389/fcell.2021.784424)
Supplement: Supplementary file 1 [file DataSheet1.PDF]

Supplementary information

Antibody	Supplier	Catalog Number	Dilution	Sample Loading (μg)
p-p38	Cell Signaling	4511	1:1000	10
p38	Life Technologies	33-8700	1:1000	10
p-Akt	Cell Signaling	9271	1:500	20
Akt	Santa Cruz	sc-1618	1:500	20
p-p65	Santa Cruz	sc-166748	1:500	20
p65	Santa Cruz	sc-8008	1:200	20
p300	Santa Cruz	sc-584	1:200	40
p-CEBPβ	Cell Signaling	3084	1:1000	20
CEBPβ	Cell Signaling	3087	1:500	20
UBR2	NOVUS Biologicals	NBP1-45243	1:1000	30
MuRF1	Santa Cruz	sc-27642	1:500	30
MHC (MF-20)	R&D Systems	MAB-4470	1:20000	10
α-Actin	ThermalFisher	MA5-12542	1:10000	10
LC3b	NOVUS Biologicals	NB100-2220	1:5000	20
p-62	MBL Life Science	PM045	1:5000	10
Vinculin	Santa Cruz	sc-73614	1:5000	20

Table S1. Information on commercial antibodies used in Western blot analysis.

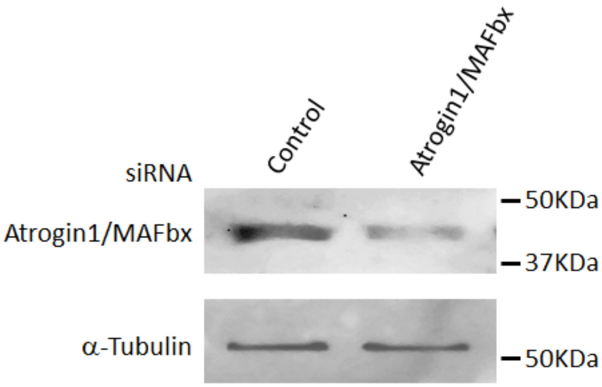

Figure S1. Validation of the specificity of custom antibody against Atrogin1/MAFbx. C2C12 myoblasts were transfected with control or Atrogin1/MAFbx-specific siRNA (SASI_Mm01_00036617, Sigma-Aldrich) using the jetPRIME reagent (Polyplus-transfection Inc., Illkirch, France) according to the manufacturer's protocol. After differentiation for 96 h, myotubes were lysed and analyzed for Atrogin1/MAFbx protein level with Western blotting using a custom antibody generated by Pocono Rabbit Farm & Laboratory (Pocono, PA) using a peptide (NILEKVVLKVLE + C and KLH conjugation) synthesized by Bon Opus Biosciences (Millburn, NJ).

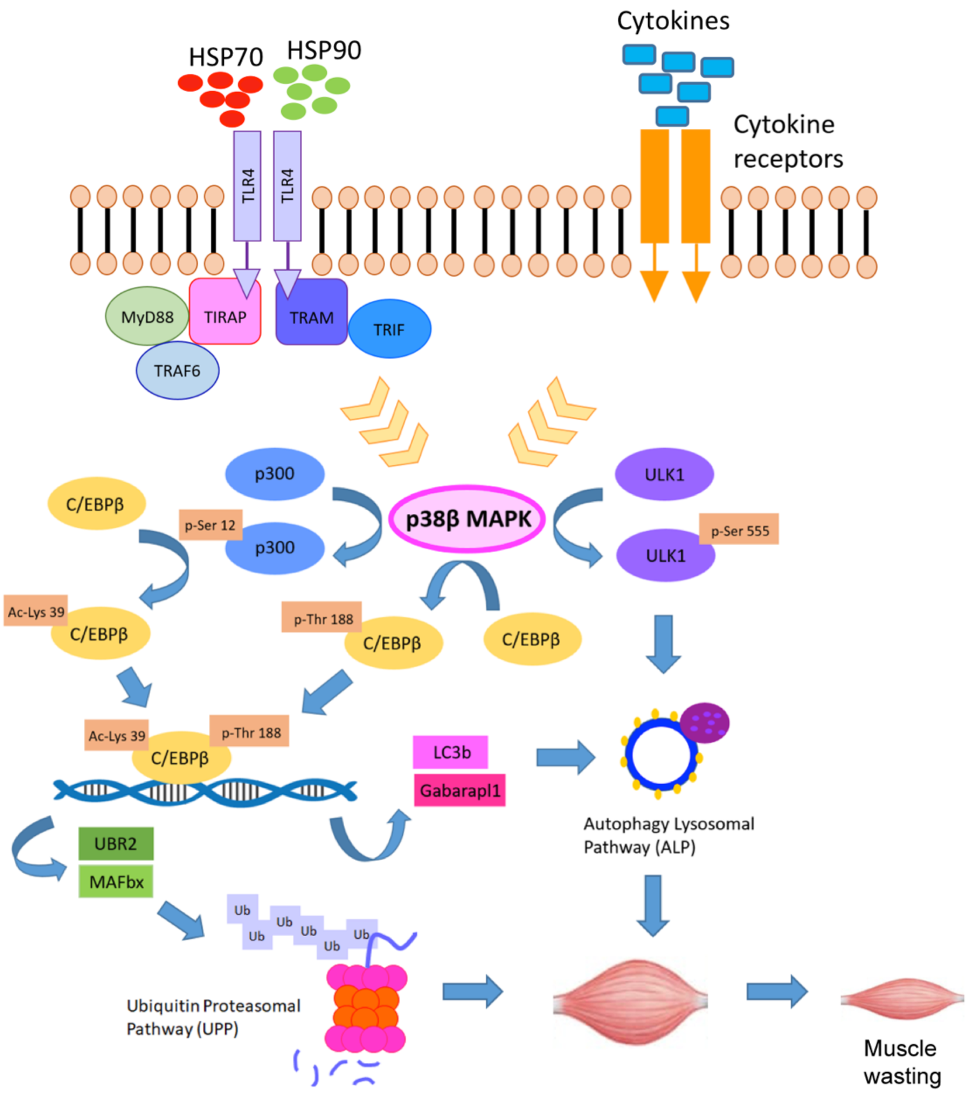

Figure S2. Schematic representation of the activation of p38β MAPK-mediated catabolic signaling pathways by circulating Hsp70/90 and cytokines that mediates cancer-induced muscle protein loss. Cachectic cancers release Hsp70 and Hsp90 into circulation to activate p38β MAPK in skeletal muscle via TLR4, which in turn activates p300 and C/EBPβ, and the latter upregulates key genes in UPP and ALP. In addition, p38β MAPK activates autophagy by directly activating ULK-1. These events cause loss of skeletal muscle proteins resulting in muscle wasting.

Systemic activation of TLR4 elevates circulation inflammatory cytokines, which also promote muscle wasting by activating p38β MAPK-mediated catabolic signaling.
